# Mechanisms of CDK4/6 Inhibitor Resistance in Luminal Breast Cancer

**DOI:** 10.3389/fphar.2020.580251

**Published:** 2020-11-16

**Authors:** Zhen Li, Wei Zou, Ji Zhang, Yunjiao Zhang, Qi Xu, Siyuan Li, Ceshi Chen

**Affiliations:** ^1^ Department of the Third Breast Surgery, The Third Affiliated Hospital of Kunming Medical University, Kunming, China; ^2^ Queen Mary Institute, Nanchang University, Nanchang, China; ^3^ Kunming Medical University Haiyuan College, Kunming, China; ^4^ Department of Molecular Biosciences, Institute of Cellular and Molecular Biology, The University of Texas, Austin, TX, United States; ^5^ Key Laboratory of Animal Models and Human Disease Mechanisms of the Chinese Academy of Sciences and Yunnan Province, Kunming Institute of Zoology, Chinese Academy of Sciences, Kunming, China; ^6^ KIZ-CUHK Joint Laboratory of Bioresources and Molecular Research in Common Diseases, Kunming Institute of Zoology, Chinese Academy of Sciences, Kunming, China; ^7^ Institute of Translation Medicine, Shenzhen Second People’s Hospital, The First Affiliated Hospital of Shenzhen University, Shenzhen, China; ^8^ Affiliated Cancer Hospital and Institute of Guangzhou Medical University, Guangzhou, China

**Keywords:** luminal breast cancer, endocrine resistance, upstream response signaling, downstream bypass signaling, CDK4/6 inhibitor

## Abstract

As a new-generation CDK inhibitor, a CDK4/6 inhibitor combined with endocrine therapy has been successful in the treatment of advanced estrogen receptor–positive (ER+) breast cancer. Although there has been overall progress in the treatment of cancer, drug resistance is an emerging cause for breast cancer–related death. Overcoming CDK4/6 resistance is an urgent problem. Overactivation of the cyclin-CDK-Rb axis related to uncontrolled cell proliferation is the main cause of CDK4/6 inhibitor resistance; however, the underlying mechanisms need to be clarified further. We review various resistance mechanisms of CDK4/6 inhibitors in luminal breast cancer. The cell signaling pathways involved in therapy resistance are divided into two groups: upstream response mechanisms and downstream bypass mechanisms. Finally, we discuss possible strategies to overcome CDK4/6 inhibitor resistance and identify novel resistance targets for future clinical application.

## Introduction

Breast cancer (BC) is a common women-related malignant tumor disease in developed countries. Estrogen receptor–positive (ER-positive) breast cancer represents approximately 70% of all BC (Goldhirsch et al., 2011; Malvezzi et al., 2013). ER-positive breast cancer can be further stratified into pathological subtypes, such as ductal or mixed ductal and lobular, mucinous, and tubular carcinomas, which are referred to as luminal breast cancer (Ignatiadis and Sotiriou, 2013). Luminal breast tumors are highly heterogeneous in terms of histology and response to treatment. Luminal A and B are two main ER-positive breast cancer subtypes, based on different gene expression profiles, prognosis, and clinical therapy responses (Sotiriou and Pusztai, 2009).

The difference between luminal A and B is mainly related to the expression of hormone receptors. Luminal B tumors have lower levels of ER expression, lower or no levels of progesterone receptor (PR) expression, but higher tumor grade and higher Ki-67–positive staining than luminal A tumors (Goldhirsch et al., 2011; Creighton, 2012). Endocrine therapy, such as ER downregulators, selective ER modulators, and aromatase inhibitors, is considered to be the primary treatment for luminal A and luminal B. However, in the clinic, the main therapy for luminal B is chemotherapy, due to the lower sensitivity of these patients to endocrine treatment or drug resistance (Rouzier et al., 2005; Ignatiadis et al., 2012). In fact, endocrine resistance is an unavoidable problem in clinical therapy of luminal tumors. Development of new therapy methods to avert endocrine resistance is an urgent challenge in clinical medicine (Anurag et al., 2020).

It is well known that the cell cycle is driven by cyclin-dependent kinases (CDKs), such as CDK4 and CDK6, which are also closely associated with tumor initiation and progression Yu et al., 2006; Choi et al., 2012). The activity of cyclin D and CDK4/6 complexes is considered to play the major role in tumor cell proliferation driven by estrogen, especially in breast cancer (Filmus et al., 1994). In recent years, it has been established that targeting the cell cycle for anticancer treatment is a rational option that could be combined with endocrine therapy.

CDK inhibitors, which target overactive CDK activities in tumor cells, have been widely used in preclinical or clinical trials. In the clinic, three CDK4/6 inhibitors, namely, palbociclib (Fry et al., 2004), ribociclib (Infante et al., 2016), and abemaciclib (Patnaik et al., 2016), have been successfully used in combination with other endocrine therapy drugs for ER-positive and human epidermal growth factor receptor-2 (HER2)–negative advanced breast cancer treatment (Ribnikar et al., 2019); in addition, significant overall survival (OS) benefits have been confirmed at ESMO2019 conference.

Despite the fact that the new guidelines for the therapy of advanced breast cancer includes a CDK4/6 inhibitor combined with endocrine treatment as the first- or second-line drug in most countries, most patients eventually develop acquired drug resistance to CDK4/6 inhibitors (Konecny et al., 2011). Several factors affect the effectiveness of CDK4/6 inhibitors, such as continuous expression of G1-S-phase cyclins and gene mutations in key cell signaling pathways (Herrera-Abreu et al., 2016). Research on the molecular mechanisms or clinical strategies to overcome CDK4/6 inhibitor resistance is ongoing (Pandey et al., 2019; Portman et al., 2019). Therefore, the major emerging consideration in treatment of advanced luminal breast cancer is now CDK4/6 inhibitor resistance.

In this review, we discuss three CDK4/6 inhibitors with different clinical trial results and various resistance mechanisms, aiming to help identify novel clinical therapeutic targets to improve endocrine therapy resistance and provide possible strategies to overcome resistance to CDK4/6 inhibitors in advanced luminal breast cancer.

### CDK4/6 Inhibitors in Luminal Breast Cancer

In malignant cells, overactive CDK activities are targeted by CDK inhibitors. The major barrier limiting CDK inhibitors from further development is the lack of selectivity, due to similar structures among CDKs (Shapiro, 2006; Michaud et al., 2010). In the meantime, some biocomputing technologies, such as computer-aided (Kalra et al., 2017) and pharmacological (Tadesse et al., 2018; Yin et al., 2018) approaches, have been employed to develop a new-generation CDK inhibitor with higher selectivity. Recently, there has been great progress in CDK inhibitor design, especially the design of CDK4/6 inhibitors, which have been successfully used in clinical trials.

ATP-binding domains are the main drug targets of CDK4/6 inhibitors to block cell cycle G1-S transition (Asghar et al., 2015). Three third-generation CDK inhibitors, palbociclib, ribociclib, and abemaciclib, have higher specificity to CDK4/6 than other members of the CDK family and have been translated into clinical use against advanced luminal breast cancer. The phase III MONALEESA-3 trial used a combination of ribociclib and fulvestrant in advanced ER+/HER2 breast cancer demonstrated an increased PFS (progression-free survival) (Slamon et al., 2018) and an improved OS compared with fulvestrant alone (Slamon et al., 2019). The phase III MONARCH-plus trial with abemaciclib and nonsteroidal aromatase inhibitor (NSAI) or fulvestrant treatment showed improved PFS in predominantly Chinese postmenopausal women with ER+/HER2 breast cancer (Jiang et al., 2019a). Moreover, in the phase III MONARCH HER trial, triple treatment with abemaciclib, trastuzumab (Herceptin), and fulvestrant showed better therapy outcomes than trastuzumab plus chemotherapy in ER+/HER2+ patients. In addition, phase II/III trials of the three CDK4/6 inhibitors in combination with letrozole, tamoxifen, fulvestrant, and herceptin in the first-/second-line setting have already been completed (Table 1).

**TABLE 1:** CDK4/6 inhibitors for the treatment of advanced luminal breast cancer in phase II/III trials.

Clinical trial	Regimen	Phase	Patients	PFS (months)	ORR	Hazard ratio		References
First line								
PALOMA-1	Letrozole + palbociclib/Letrozole	Ⅱ	165	10.2 vs. 20.2	39 vs. 55%	0.49		Finn et al. (2015)
PALOMA-2	Letrozole + palbociclib/Letrozole	Ⅲ	666	14.5 vs. 24.8	44 vs. 55%	0.58		Finn et al. (2016)
MONALEESA-2	Letrozole ± ribociclib	Ⅲ	668	14.7 vs. 26.0	37 vs. 53%	0.57		Hortobagyi et al. (2018)
MONARCH-3	NSAI ± abemaciclib	Ⅲ	493	14.7 vs. 28.2	44 vs. 59%	0.54		Goetz et al. (2017)
MONALEESA-7	NSAI/Tamoxifen + OFS ± ribociclib	Ⅲ	672	13.0 vs. 23.8	36 vs. 51%	0.55		Tripathy et al. (2018)
Second line								
PALOMA-3	Fulvestrant ± palbociclib	Ⅲ	521	4.6 vs. 9.5	11.1 vs. 25%		0.46	Cristofanilli et al. (2016)
MONARCH-1	Abemaciclib monotherapy	Ⅱ	132	6.0	20%		—	Dickler et al. (2017)
MONARCH-2	Fulvestrant ± abemaciclib	Ⅲ	669	9.3 vs. 16.4	21 vs. 48		0.55	Sledge et al. (2017)
MONALEESA-3	Fulvestrant ± ribociclib	Ⅲ	725	12.8 vs. 20.5	29 vs. 41%		0.59	Slamon et al. (2018)
MONARCH-plus	NSAI ± abemaciclib	Ⅲ	306	14.73 vs. NE	30.3 vs. 56%		0.499	Jiang et al. (2019a)
	Fulvestrant ± ribociclib	Ⅲ	157	5.59 vs. 11.47	7.5 vs. 38.5%		0.376	
MONARCH HER	Herceptin + abemaciclib + fulvestrant	Ⅲ	79	8.32 vs. 0.65 vs. 5.69	32.9 vs. 13.9 vs. 13.9%		0.673	Tolaney et al. (2019)
	Herceptin + abemaciclib	Ⅲ	79				0.943	
	Herceptin + chemo	Ⅲ	79				—	

PFS, progression free survival; NSAI, nonsteroidal aromatase inhibitors; OFS, ovarian function suppression; ORR, objective response rate; NE indicates that the value could not be estimated.

### Resistance Mechanisms of CDK4/6 Inhibitor

**FIGURE 1:**
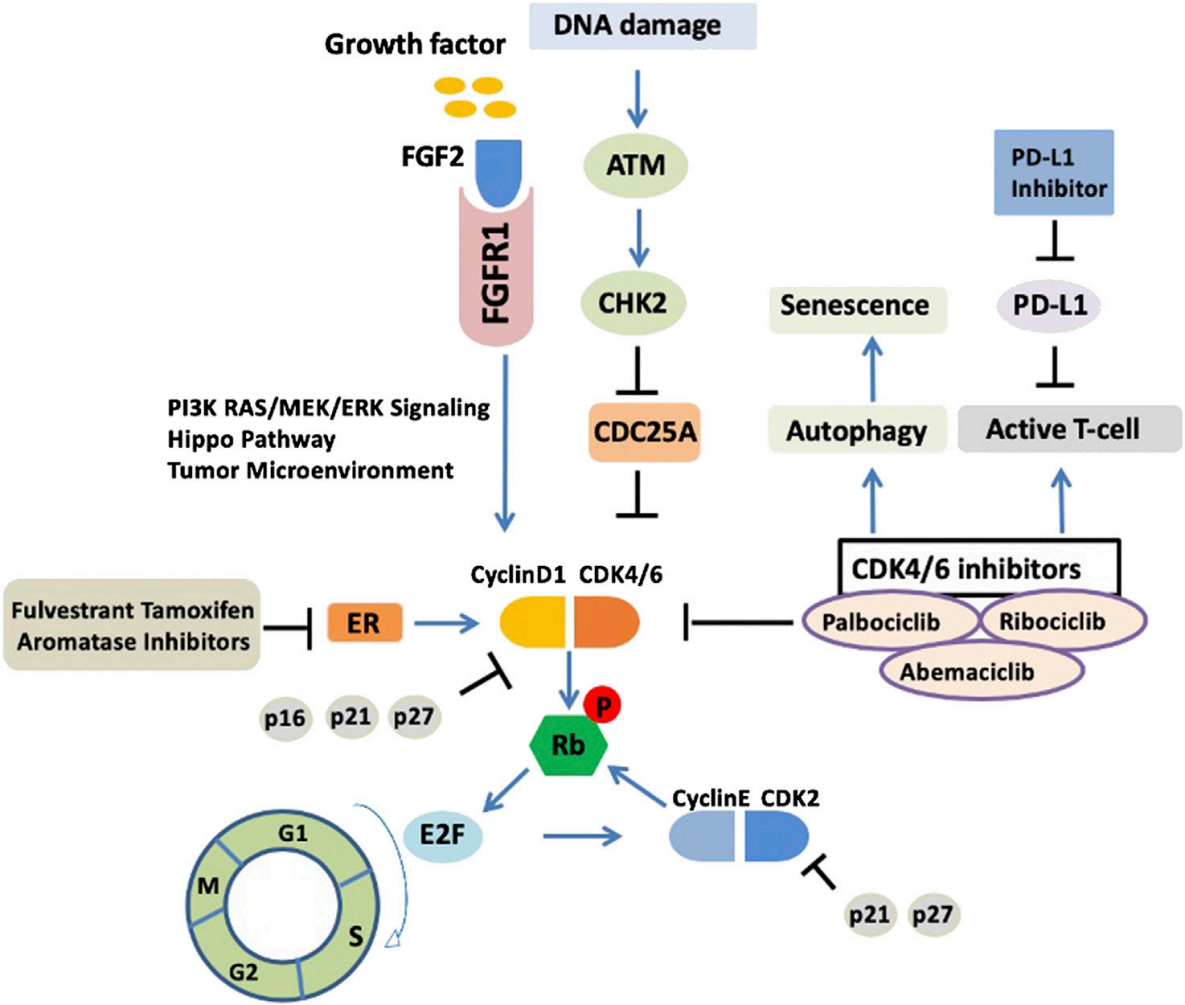
Upstream response and downstream bypass signaling mechanisms of CDK4/6 inhibitor resistance. Current molecular mechanisms of CDK4/6 inhibitor resistance are highlighted. ER, estrogen receptor; CDK, cyclin dependent kinases; ATM, ataxia telangiectasia mutated; CHK2, checkpoint kinase 2; Rb, retinoblastoma protein.

CDK4/6 inhibitors are not a panacea due to the therapy resistance. It has been reported in the PALOMA-2 trial that more than 30% patients experienced recurrence of their cancer within 2 years of CDK4/6 inhibitor treatment (Finn et al., 2016), indicating that palbociclib combined with endocrine therapy may affect CDK inhibitor sensitivity and allow tumor cells to return to a proliferative phenotype. However, whether the mechanism of endocrine therapy resistance is associated with the inhibition of cell cycle or activation of other “bypass” signaling pathways is not fully understood. We summarized the molecular mechanisms of CDK4/6 inhibitor resistance below (Figure 1).

### CyclinD1-CDK4/6–Rb Pathway

Upregulation of cyclin–CDK activity promotes the cell cycle and proliferation (Hanahan and Weinberg, 2011). The retinoblastoma (Rb) protein acts as a gatekeeper to prevent the cell cycle from progressing from G1 phase into S phase. CDK4/6 forms heterodimers with D-type cyclins (particularly D1) to phosphorylate Rb. As a result, Rb protein phosphorylation leads to the release of transcription factor E2Fs, which activates the DNA transcriptional program for cell cycle G1/S.

In luminal breast cancer, the development of resistance to endocrine therapy is associated with the function and integrity of Rb (Musgrove et al., 2011). Fortunately, the low incidence of Rb gene deletion/mutation (3.9%) in luminal-like breast tumors offers the possibility for CDK4/6 inhibition (Ciriello et al., 2015). This viewpoint has been solidified by a study that showed that the effects of clinical therapy in luminal breast cancer were not sensitive to palbociclib when Rb expression is absent (Dean et al., 2012). In addition, the function of Rb can also be regulated by E2F transcriptional factors. Malorni et al. indicated that the expression of both E2F1 and E2F2 could cause loss of Rb and predict the sensitivity of cell lines to palbociclib in luminal breast cancer (Malorni et al., 2016).

### CDK4/6 Overexpression

Overexpression of CDK4 or CDK6 is the main mechanism of resistance to CDK4/6 inhibitors. Studies have shown that increased expression of CDK6 reduced the response of CDK4/6 inhibitors in luminal cell line models. At the same time, knockdown of CDK6 rescued the therapy sensitivity, which indicated that CDK6-mediated drug resistance may be independent of CDK4 expression (Yang et al., 2017). In addition, either high or low expression of CDK4 has been detected in CDK4/6 inhibitor–resistant breast cancer cells (Bollard et al., 2017). Therefore, whether the expression of CDK4 is associated with CDK4/6 inhibitor resistance requires further investigation.

### p16 Amplification

As a member of the INK4 family, p16 is a natural inhibitor of CDK4 and plays a vital role in the regulation of the cell cycle (Serrano et al., 1993). In general, p16 severs as a tumor suppressor and targets the CDK4/6 complex in dysregulatory cells depending on the function of Rb (Medema et al., 1995). For example, Dean JL et al. reported that the resistance to CDK4/6 inhibitors was caused by the absence of Rb, regardless of p16 expression (Dean et al., 2012). On the other hand, the expression level of p16 affected the effectiveness of CDK4/6 inhibition. Overexpression of p16-mediated resistance to CDK4/6 inhibitors in the absence of Rb (Witkiewicz et al., 2011) and low expression of p16 did not rescue the clinical benefit in Rb-positive luminal breast cancer patients in the phase II palbociclib monotherapy trial (DeMichele et al., 2015). The potential mechanism is that p16 overexpression suppresses the activity of CDK4 and expression of cyclin D1 (Witkiewicz et al., 2011), which are the main targets of CDK4/6 inhibitors, thus leading to reduced or no effects of CDK4/6 inhibition (Elvin et al., 2017). Whether p16 amplification and loss of Rb work together in CDK4/6 inhibitor resistance is not clearly understood. Further studies revealing the mechanistic association between p16 and Rb might be beneficial to avert acquired resistance to CDK4/6 inhibitors.

### ATM-CHK2 Activation

Deficiency of mismatch repair may lead to the endocrine therapy resistance in luminal breast cancer through the abrogation of CHK2-mediated inhibition of CDK4. A recent study showed that defects in single-strand break repair in luminal breast cancer can drive endocrine therapy resistance and is closely associated with the ATM-CHK2-CDC25A pathway (Anurag et al., 2018). ATM, as a DNA damage sensor, activates CHK2, which in turn phosphorylates CDC25A at S123 for degradation. Importantly, as a phosphatase, CDC25A could inhibit the phosphorylation of CDK4/6. The CDK4/6 complex activity could be reactivated with the “on state” of CDC25A. Therefore, the cross talk between the CDK4/6–Rb and ATM–CHK2–CDC25A axes is very important. Moreover, recently, Haricharan et al. demonstrated that for the efficacy of endocrine agents in luminal tumors, both ATM and CHK2 are required; inactivation of either of these negative cell cycle regulators prevents cell cycle arrest upon ER inhibition (Haricharan et al., 2017).

### Loss of ER Expression

In luminal breast cancer, activation of ER is the major driver of CDK4/6. Selective ER-related endocrine therapy, such as ER downregulators (fulvestrant), ER modulators (tamoxifen), and aromatase inhibitors (AIs), have been combined with CDK4/6 inhibitors and broadly used in the treatment of advanced ER-positive breast cancer. The expression level of cyclin D1 could be upregulated by ER (Du et al., 2014). Resistance to CDK4/6 inhibitors may be related to the decrease in cyclin D1 due to the loss of ER (Gong et al., 2017b). For instance, resistance to the CDK4/6 inhibitor abemaciclib occurred in preclinical trials and was associated with the loss of cyclin D1 and ER/PR expression. In addition, studies showed that CDK6 overexpression diminished the responsiveness to ER antagonism and mediated the resistance to CDK4/6 inhibitors by decreasing the expression of ER and PR (Yang et al., 2017). Moreover, tumor biopsy specimens from patients associated with changes in ER/PR levels showed resistance to CDK4/6 inhibitors mediated by low ER/PR expression. Moreover, it also has been indicated that luminal tumors are resistant to endocrine therapy when they have an activating *ESR1* mutation; however, CDK4/6 inhibitors take effect regardless of *ESR1* mutation status (Fribbens et al., 2016).

### Activation of PI3K–AKT–mTOR Signaling

The PI3K–AKT–mTOR signaling pathway is involved in tumor cell growth, survival, and metastasis. In luminal breast cancer, ER transcriptional activity could be enforced by the activation of PI3K–AKT–mTOR signaling, which drives endocrine therapy resistance (Miller et al., 2011). Furthermore, activation of the PI3K–AKT–mTOR pathway can also promote the stability of the CDK4/6 complex, thus reversing the effects of CDK4/6 inhibition (Miller et al., 2011). A recent study indicated that loss of PTEN expression could mediate CDK4/6 inhibitor resistance by increasing AKT activation and decreasing the expression of p27, which leads to the excessive activation of CDK4 and CDK2 (Costa et al., 2020). CDK4 in lysosome activates mTORC1 and is also associated with cancer progression (Martinez-Carreres et al., 2019). Moreover, it has been reported that CDK4/6 inhibitors preferred to activate PI3K–AKT–mTOR pathway than ER signaling (Takeshita et al., 2018), with the reactivation of E2F (Jansen et al., 2017). Therefore, the therapeutic trial, endocrine therapy backbone combined with PI3K and mTOR inhibition, and CDK4/6 inhibitors can be combined.

Strategies that inhibit PI3K and mTOR activities have been shown to restore sensitivity to endocrine therapy. Everolimus, a mTOR inhibitor, was the first drug developed to overcome endocrine therapy resistance in combination with AI (Pronzato, 2017). Some other mTORC1/2 inhibitors also restored the sensitivity of CDK4/6 inhibitors in resistant cells by suppressing Rb phosphorylation (Michaloglou et al., 2018). PI3K inhibitors, such as alpelisib, combined with fulvestrant prolonged PFS among patients with mutated PIK3CA in advanced luminal breast cancer who had previously received endocrine therapy (Andre et al., 2019). In addition, PI3K inhibitors have been implicated in the prevention of early CDK4/6 inhibitor adaptions by decreasing the expression of cyclin D1 (Herrera-Abreu et al., 2016). In the future, a combination of a PI3K–AKT–mTOR pathway inhibitor and a CDK4/6 inhibitor may be a valuable therapeutic strategy.

### Upregulation of FGFR Pathway

The fibroblast growth factor receptor (FGFR) pathway is involved the proliferation and survival in luminal breast cancer (Sahores et al., 2018). Like other mitogenic pathways, FGFR is relevant in the crosslinking of cyclin D and CDK4/6. Of the five FGFRs, FGFR1 is associated with CDK4/6 inhibitor resistance. FGFR1 activates the PI3K–AKT–mTOR and RAS–MEK–ERK signaling pathways (Turner et al., 2010). In the clinic, FGFR1 overexpression mediated resistance to palbociclib or ribociclib when combined with endocrine therapy (fulvestrant) (Formisano et al., 2017). This could be reversed by the FGFR tyrosine kinase inhibitor (TKI) lucitanib (Formisano et al., 2019). FGFR2 amplification has also been reported in metastatic luminal breast cancer and the response to an mTOR inhibitor (Wein et al., 2017). In addition, FGF2 could also activate FGFR signaling and mediate endocrine therapy resistance in preclinical research (Turner et al., 2010). A previous study showed that the FGFR2 inhibitor formononetin had a strong inhibitory effect on angiogenesis and tumor growth (Wu et al., 2015). Therefore, targeting FGFR1/2 in luminal breast cancer may be a viable option combined with the inhibition of CDK4/6 to overcome CDK4/6 inhibitor resistance.

### Alterations of Hippo Pathway

The Hippo pathway is closely related to the development and progression of breast cancer and has emerged as a linchpin in breast cancer therapy resistance (Gujral and Kirschner, 2017) (Shi et al., 2015). Hippo pathway effectors, such as YAP, TAZ, and TEAD, have been employed as drug targets to hit other signaling pathways (Dey et al., 2020). In ovarian cancer, YAP expression is associated with PI3K inhibitor resistance (Muranen et al., 2016). TEADs have also been shown to be a mediator of CDK6 induction (Xie et al., 2013). Importantly, alterations in the Hippo pathway are related to CDK4/6 inhibitor resistance. In the latter clinical case, loss of *FAT1* is associated with CDK4/6 inhibitor resistance caused by YAP/TAZ nuclear localization and CDK6 overexpression in ER-positive breast cancer (Li et al., 2018). Therefore, targeting the Hippo pathway offers a new therapeutic strategy against CDK4/6 inhibitor resistance.

### Downstream Bypass Signaling Mechanisms

The molecular mechanisms responsible for resistance to CDK4/6 inhibitors are diverse and complicated, and the current knowledge is far from complete. Recently, several new “bypass” signaling pathway mechanisms on CDK4/6 inhibitor adaption have been discovered.

### Activation of CDK2 Signaling

Cyclin E–CDK2 complexes can also inactivate Rb by phosphorylating Rb and releasing transcriptional factor E2F to initiate the cell cycle. However, as the “second wave” that phosphorylates Rb, the efficiency of this process is subsequent to CDK4/6 complexes. Excessive activation of the CDK2 pathway mediates resistance to CDK4/6 inhibitors because released E2F reverse targets cyclin E2, stabilizing the cyclin E2–CDK2 complexes and reducing CDK4/6 inhibition (Caldon et al., 2009). The abnormal expression of cyclin E1/2-CDK2 and persistent activation of E2F are associated with resistance to CDK4/6 inhibitors (Taylor-Harding et al., 2015). For instance, *CCNE1* gene amplification also induces resistance in the CDK4/6 single agent model; *CCNE2* gene amplification has been found in patients in whom palbociclib treatment failed (Hortobagyi et al., 2016). Moreover, in the clinic, lower *CCNE1* messenger RNA expression is often associated with improved palbociclib efficacy in ER-positive metastatic breast cancer (Turner et al., 2019). Activity of cyclin E1–CDK2 complexes could be suppressed by p21^Waf1/Cip1^ and p27^Kip1^ (Martin et al., 2017); therefore, the development of CDK2 inhibitors have the potency and advantage as bypass signals to reduce CDK4/6 inhibitor resistance by the inhibition of cyclin E1/2–CDK2 (Caldon et al., 2012).

### Autophagy

Autophagy is generally thought of as a cell survival mechanism. The activation of autophagy induces cell cycle arrest and cell senescence (Glick et al., 2010). Targeting autophagy is an available strategy for novel drug development and tumor treatment. Autophagy inhibition is relevant to the efficacy of anti–breast cancer drugs (Chittaranjan et al., 2014). An accumulation of evidence suggests that autophagy activation is involved in resistance to CDK4/6 inhibitors. Studies have shown that breast cancer cells activate autophagy in response to palbociclib, possibly through the inhibition of cyclin D1 expression, and the combination of autophagy and CDK4/6 inhibitors induces irreversible growth inhibition and senescence *in vitro* (Vijayaraghavan et al., 2017b). More work is being done to increase the efficacy of CDK4/6 inhibitors by inhibiting autophagy, which may help avert CDK4/6 inhibitor resistance.

### Immune Evasion

The adaptive immune response plays a role in CDK4/6 inhibitor efficacy. CDK4/6 inhibitors promote tumor immunogenicity, and the effects of CDK4/6 inhibitors targeting both tumor T cells and regulatory T cells are associated with reduced activity of E2F transcription factors and DNA methyltransferase (Goel et al., 2017). In addition, CDK4/6 inhibitors enhance antitumor immunity by increasing T-cell activation and promoting T cells to kill tumor cells (Deng et al., 2018). Moreover, immunotherapeutic approaches combined with CDK4/6 inhibitors could achieve better therapeutic effects. CDK4/6 inhibitors increase the expression of PD-L1 (programmed cell death ligand 1), thus inducing the inflammatory microenvironment and improving tumor immunogenicity (Minton, 2017; Schaer et al., 2018). Therefore, CDK4/6 inhibitors combined with a PD-L1 immune checkpoint inhibitor can improve the effect of tumor immunotherapy. Currently, there are several ongoing clinical trials of immune checkpoint antibodies, including pembrolizumab and avelumab (Anurag et al., 2020). However, immune evasion or alterations in the immune microenvironment eventually leads to CDK4/6 inhibitor resistance (Goel et al., 2017; Teh and Aplin, 2019). In terms of mechanism, immune evasion may be associated with the abnormal expression of immune-related regulators, such as IFN-α and IFN-β, and change in tumor microenvironment of CDK4/6 inhibitor–resistant breast tumors (Vijayaraghavan et al., 2017a). Future investigations using tumor-infiltrating lymphocyte analyses are needed to better understand CDK4/6 inhibitor resistance mechanisms of immune evasion.

### Epigenetic Alterations

Histone deacetylases (HDACs) can increase CDK4/6 inhibition efficacy and mediate cell cycle arrest by upregulating p21 expression in CDK4/6 inhibitor resistant tumors (Lee et al., 2018). Even though the mechanism is not very clear, HDAC inhibition works synergistically with CDK4/6 inhibitors in luminal breast cancer. Cornell et al. demonstrated that miR-432-5p–mediated suppression of the TGF-β signaling pathway via SMAD4 knockdown and increased CDK6 expression, thus conferring transmissible and reversible CDK4/6 inhibitor adaptation (Cornell et al., 2019). In addition, a recent study showed that LncRNA TROJAN could mediate resistance to CDK4/6 inhibitors by increasing CDK2 activation in ER+ breast cancer (Jin et al., 2020). Analysis of patient plasma exosomes may identify emerging resistance mechanisms.

### Strategies to Overcome CDK4/6 Inhibitor Resistance

In the clinic, treatment effectiveness is based on the improved survival of patients. Currently, endocrine targeted therapy and chemotherapy are common options for the treatment of luminal breast cancer. CDK4/6 inhibitors have been used in advanced ER-positive breast cancer patients with antimitosis, but they eventually develop resistance to the CDK4/6 inhibitors (Franco et al., 2014; Yoshida et al., 2016). In the past 5 years, endocrine therapy combined with PI3K and mTOR inhibitors and CDK4/6 inhibitors has gradually become a new therapeutic strategy. Several studies have confirmed that CDK4/6 inhibitors combined with PI3K inhibitors (Vora et al., 2014) or mTORC1/2 inhibitors could reverse resistance (Michaloglou et al., 2018). Furthermore, studies have shown that CDK4/6 inhibitors may increase tumor immunogenicity, which provides a rationale for combination regimens composed of CDK4/6 inhibitors and immunotherapies. Therefore, CDK4/6 inhibitors combined with other clinical therapies might be a cautious approach to overcome therapy resistance. We summarized possible strategies to overcome resistance to CDK4/6 inhibitors in Table 2.

**TABLE 2:** Possible strategies to overcome resistance to CDK4/6 inhibitors in ER-positive BC.

Resistance study	Potential mechanisms	Possible strategies	References
Cell cycle genes	Rb, cyclin D1, cyclin E	Intact Rb, *CCNE1* amplification	Turner et al. (2019)
	CDK4, CDK6	Knockdown of CDK4 and CDK6	Yang et al. (2017)
	*p16, p21, p27*	Intact Rb and knockdown of *p16*	Dean et al. (2012), Elvin et al. (2017)
Crosstalk pathways	ATM-CHK2	ATM inhibitor Ku60019	Haricharan et al. (2017), Anurag et al. (2018), Lang et al. (2018)
	PI3K/AKT/mTOR	PI3K-AKT-mTOR inhibitors	Costa et al. (2020)
	ER	Selective ER-related endocrine therapy	Fribbens et al. (2016)
	FGFR	FGFR2 inhibitor formononetin	Wu et al. (2015)
	Hippo	*FAT1*, verteporfin, CA3, VGLL4 peptide	Liu-Chittenden et al. (2012), Li et al. (2018), Song et al. (2018), Smith et al. (2019)
	CDK2	Flavopiridol, AT7519, dinaciclib	Tan et al. (2004), Squires et al. (2009), Parry et al. (2010)
	Autophagy	NAPI, ATG7, chloroquine	Liang et al. (2016), Gong et al. (2017a), Cui et al. (2018)
Combination therapy	Endocrine therapy	Fulvestrant, tamoxifen and AI	Turner et al. (2017)
	PI3K/mTOR inhibitor	Alpelisib, everolimus	Pronzato (2017), Andre et al. (2019)
	Immune checkpoint inhibitor	Pembrolizumab, atezolizumab, nivolumab	Kok et al. (2018), Schmid et al. (2019), Schneeweiss et al. (2019)
	Epigenetic inhibitor	Romidepsin, vorinostat, tucidinostat	Robertson et al. (2013), Bian et al. (2018), Jiang et al. (2019b)

NAPI, nanoparticle autophagy inhibitors.

### Potential Biomarkers for Predicting CDK4/6 Inhibitor Resistance

Whether CDK4/6 inhibition is truly suitable for patients with advanced ER-positive breast cancer and whether resistance develops are being studied in a number of preclinical studies and models. Rb may be a biomarker. It has been demonstrated that fully functional Rb is required for the effective use of CDK4/6 inhibitors in the clinic (Karakas et al., 2016). However, not all Rb+/ER+ patients would benefit from CDK4/6 inhibitor therapy, even though the mutation of Rb is very rare (3.9%) in ER-positive breast cancer. The utility of Rb as biomarker combined with low-molecular-weight cyclin E1 (LMWE) is associated with CDK4/6 inhibitor sensitivity (Hunt et al., 2017). A cohort of 109 patients with Rb-/LMWE+ had shorter PFS when treated with palbociclib plus endocrine therapy (Vijayaraghavan et al., 2017b). Although cyclin D1 plays a vital role in CDK4/6 inhibition, unfortunately, *CCND1* amplification as single biomarker for CDK4/6 inhibitor sensitivity needs to be refined further. In the PALOMA-1 study, patients treated with palbociclib plus letrozole had no beneficial outcomes regardless of *CCND1* status (Finn et al., 2015). Moreover, CDK4 phosphorylation status shows the potential as a biomarker to predict the sensitivity to palbociclib but needs further clinical observation (Raspe et al., 2017).

## Conclusion

The development of CDK4/6 inhibitors has been a significant advancement in luminal breast cancer therapy. In other breast cancer subtypes, such as triple negative breast cancer, clinical trials of CDK4/6 inhibitors in combination with anti-androgen inhibitors are still ongoing. However, resistance to CDK4/6 inhibitors in clinical treatment is an unavoidable problem. Although CDK4/6 inhibitor resistance has been well investigated and different mechanisms have been revealed, systematic and comprehensive clinical trials are required to develop new strategies to overcome CDK4/6 inhibitor resistance. Therefore, further efforts to investigate much more precise resistance mechanisms to CDK4/6 inhibitors or to develop more successful CDK inhibitors are needed in order to explore new therapeutic approaches to avert or overcome resistance.

## Author Contributions

ZL: conceptual design and article writing; WZ: work drawing; JZ: conceptual design; YZ: make keyword tables; QX: work drawing; SL: conceptual design and article writing; CC: conceptual design and article writing

## Funding

This study was supported in part by grants from the National Key R&D Program of China [2018YFC2000400], the National Nature Science Foundation of China [81830087, U1602221, 31771516], Science and Technology Innovation Team of Yunnan Province [2018HC002, 2019HC005], Basic Research Program of Yunnan Province [2019FE001(-309)], and Research Institutions attached to the Health and Family Planning Commission of Yunnan Province [2017NS180].

## Conflict of Interest

The authors declare that the research was conducted in the absence of any commercial or financial relationships that could be construed as a potential conflict of interest.
